# Clinical Impact of Multiparametric Contrast‐Enhanced Dual‐Energy Computed Tomography in Arthritis Imaging: A Prospective Single‐Center Study

**DOI:** 10.1002/art.43270

**Published:** 2025-08-09

**Authors:** Sevtap Tugce Ulas, Jürgen Mews, Sarah Ohrndorf, Robert Biesen, Katharina Ziegeler, Edgar Wiebe, Fabian Proft, Udo Schneider, Denis Poddubnyy, Torsten Diekhoff

**Affiliations:** ^1^ Department of Radiology Charité – Universitätsmedizin Berlin, Campus Mitte, Humboldt – Universität zu Berlin, Freie Universität Berlin Berlin Germany; ^2^ Canon Medical Systems, Europe BV Amstelveen The Netherlands; ^3^ Department of Rheumatology Charité – Universitätsmedizin Berlin, Campus Mitte, Humboldt – Universität zu Berlin, Freie Universität Berlin, Berlin, Germany, and Department of Gastroenterology, Hepatology, Infectious Diseases, and Rheumatology, Klinikum Ernst von Bergmann Potsdam Potsdam Germany; ^4^ Department of Rheumatology Charité – Universitätsmedizin Berlin, Campus Mitte, Humboldt – Universität zu Berlin, Freie Universität Berlin Berlin Germany; ^5^ University of California San Francisco; ^6^ Department of Gastroenterology Infectious Diseases, and Rheumatology, Charité – Universitätsmedizin Berlin, Campus Benjamin Franklin, Humboldt – Universität zu Berlin, Freie Universität Berlin Berlin Germany; ^7^ Department of Gastroenterology Infectious Diseases, and Rheumatology, Charité – Universitätsmedizin Berlin, Campus Benjamin Franklin, Humboldt – Universität zu Berlin, Freie Universität Berlin, Berlin, Germany, and Division of Rheumatology, Department of Medicine, University Health Network and University of Toronto Toronto Ontario Canada

## Abstract

**Objective:**

We aimed to evaluate the influence of contrast‐enhanced dual‐energy computed tomography (CE‐DECT) in detecting and differentiating rheumatic joint diseases of the hand.

**Methods:**

In this prospective study, patients with suspected arthritis of the hand were investigated consecutively alongside the standard clinical procedure. CE‐DECT with sequential rotations was performed in all patients before and 3‐minutes after weight‐adapted contrast agent application. Reconstructions included two‐material decomposition for tophus imaging, virtual noncalcium for bone marrow edema, and CT subtraction for soft‐tissue inflammation. All postprocessed images and original CT reconstructions were rated by two radiologists in consensus to generate an imaging diagnosis. Imaging findings in CE‐DECT were juxtaposed with the initial and final evaluations by referring rheumatologists. This evaluation focused on surrogate performance criteria, emphasizing the added diagnostic value of CE‐DECT in detecting specific imaging biomarkers associated with various arthritic pathologies. A subsequent survey assessed CE‐DECT's diagnostic utility and impact on patient management, rated on a 1 to 10 scale. Descriptive statistics were employed.

**Results:**

Overall, 136 patients were included in the analysis. In 119 patients (87.5%), the CE‐DECT findings agreed with the final diagnosis. In 67 patients (49.2%), the diagnosis was changed following CE‐DECT. Rheumatologists rated CE‐DECT's diagnostic utility at a mean ± SD of 8.5 ± 2.1 and its contribution to patient management at 8.4 ± 1.8.

**Conclusion:**

CE‐DECT showed high value for diagnosis and management of patients with suspected inflammatory arthritis. Its diverse diagnostic capabilities suggest that it can develop an important addition to current clinical workup pathways.

## INTRODUCTION

Imaging plays a crucial role in the diagnosis of arthritis, particularly because clinical differentiation of joint complaints is often challenging.^1^ Early diagnosis is of paramount importance, as timely and accurate treatment can prevent irreversible damage to bones and joints, ultimately preserving joint function and enhancing patients’ quality of life.^2^, ^3^, ^4^ To facilitate this, it is essential to use standardized imaging techniques that are both readily accessible and capable in the differential diagnostic process, thereby contributing to a well‐founded indication for anti‐inflammatory therapies.^5^

Recent advancements in computed tomography (CT) and in its related postprocessing techniques have significantly enhanced its utility in clinical practice, allowing comfortable scan positioning for patients with comparably low radiation exposure in peripheral joint imaging. This opens new possibilities for information acquisition.^6^ CT has become the gold standard for evaluating bony changes such as erosions and osteophytes, which are critical for distinguishing between different forms of arthritis, including osteoarthritis (OA).^3^, ^7^ One established procedure in clinical routine is the detection of tophi using dual‐energy CT (DECT), which not only aids in diagnosis but also provides the possibility to assess the therapeutic response by quantifying tophus burden.^8^, ^9^, ^10^

Furthermore, DECT offers diverse capabilities that allow for the detection of acute soft‐tissue inflammation using intravenous contrast agent application, such as synovitis and tenosynovitis, with diagnostic performance comparable to magnetic resonance imaging (MRI) and ultrasound.^6^, ^11^, ^12^, ^13^, ^14^ For getting these insights, techniques such as CT subtraction (CT‐S),^11^ requiring pre‐ and postcontrast imaging and an integrated registration process for motion correction, and iodine maps^12^, ^13^ can be applied. These methods contribute significantly to therapeutic management and enhance the understanding of various pathobiomechanical changes associated with different inflammatory joint diseases.^15^ An important advantage of contrast‐enhanced DECT (CE‐DECT) lies in its ability to expand the differential diagnostic spectrum, for instance, by reliably detecting urate or calcium pyrophosphate dehydrate deposition disease (CPPD) crystals, which is not sufficiently possible with MRI or ultrasound. So far, bone marrow edema could only be visualized using MRI, which indicates impending structural damage in the context of arthritis imaging.^16^ Therefore, its detection plays a crucial role in clinical practice, as it often necessitates adjustments to therapy. DECT facilitates the calculation of virtual noncalcium (VNCa) maps through three‐material differentiation, allowing for the visualization of bone marrow edema.^17^, ^18^

Previous studies have demonstrated that CE‐DECT enables the detection of essential imaging parameters in arthritis.^6^, ^12^ The comprehensive information about active bone and soft‐tissue inflammation, structural bone lesions, and crystal arthropathies and its high degree of standardization and patient comfort suggest that CE‐DECT could be a versatile imaging technique for assessing different arthritides. Therefore, the aim of this study was to evaluate the influence of CE‐DECT in detecting and differentiating peripheral rheumatic joint diseases to determine its diagnostic value.

## MATERIALS AND METHODS

### Patients

This study was designed as a prospective single‐center study and included patients with suspected arthritis of the hand who presented to the rheumatology outpatient or inpatient clinic of our hospital between May 2022 and September 2024. Patients were eligible for inclusion following a conventional clinical evaluation by board‐certified referral rheumatologists. Inclusion criteria are suspected peripheral joint arthritis, patients aged >18 years, and patients with completed standard workup. The standard evaluation included clinical examination, laboratory tests, and the use of imaging modalities such as x‐ray, ultrasound, and MRI as deemed necessary by the rheumatologist. Patients with contraindication to the contrast agent (known allergic reactions to iodine, reduced kidney function, and hyperthyroidism), patients who were pregnant, and patients not able to give informed consent were excluded from the study.

### Ethical approval and patient and public involvement

All patients gave written informed consent before enrollment. The investigations were approved by the local ethics committee (EA1_183_21) and the Federal Office for Radiation Protection (ZD 3‐22464/2021‐285‐G). All procedures performed in studies involving human participants were in accordance with the ethical standards of the institutional and/or national research committee and with the 1964 Declaration of Helsinki and its later amendments or comparable ethical standards. There was no specific patient or public involvement in this investigation.

### Imaging procedure

All patients underwent CE‐DECT of the clinical dominant hand alongside the standard clinical evaluation. CE‐DECT was performed on a 320‐row single‐source CT scanner (Canon Aquilion ONE Vision or Aquilion ONE Prism, Canon Medical Systems) with a sequential volume acquisition or rapid kVp switching acquisition of two different energy datasets (135 and 80 kVp) depending on the scanner. Scans were performed after Scanogram (two‐dimensional image used for planning) before and 3 minutes after contrast injection of body weight–adjusted contrast medium 1 mL/kg Ultravist 370 (Bayer), Accupaque 350 (GE Healthcare) or Imeron 350 (Bracco Imaging) with 16‐cm z‐axis coverage without table movement. The rotation time was 0.275 or 0.5 seconds depending on the CT machine. The radiation exposure (estimated effective dose) was calculated using the overall dose‐length product and a conversion coefficient of 0.0008 (mSv × mGy^−1^ × cm^−1^).^19^

### Image processing and reconstruction algorithms

The DECT images were reconstructed using a soft‐tissue kernel without beam hardening compensation and a sharp bone kernel with a thickness of 0.5 mm on the coronal, transversal, and sagittal planes using iterative reconstructions (AIDR‐3D standard; Vision) and artificial intelligence reconstructions (spectral AICE, Prism), respectively. Further image postprocessing was conducted using the proprietary reconstruction module on the CT console (SV 6.0) or the Vitrea enterprise SV 7.15 (Vital Images) generating high‐resolution multiplanar reconstructions for morphologic assessments, two‐material decomposition for tophus imaging, VNCa for bone marrow edema, and CT‐S for soft‐tissue inflammation.

#### Tophus imaging

For both precontrast and postcontrast DECT scans, a two‐material decomposition algorithm (Dual‐Energy Composition Analysis, version 6.0, Canon Medical Systems, or using Vitrea enterprise 7.15 software) was applied separately to detect uric acid. This method differentiates between urate and soft tissue by exploiting their distinct dual‐energy attenuation characteristics, as established in previous studies.^20^, ^21^

#### VNCa

In contrast, three‐material decomposition was used to generate VNCa images based on the precontrast DECT datasets on the CT console (dual‐energy image view and dual‐energy raw data analysis, version 6.0, Canon Medical Systems) or using the Vitrea enterprise 7.15 software. This technique allows for the separation of calcium, fat, and water by defining specific attenuation values. Material formulas were −136/−106 HU (80 kVp/135 kVp) for fat and 0/0 HU (80 kVp/135 kVp) for water applying a gradient of 0.69 for calcium. The resulting VNCa images effectively suppress bone mineral to enhance the visibility of bone marrow alterations.

#### Pre‐ and postcontrast CT

Pre‐ and postcontrast CT images at 80 kVp were postprocessed in soft‐tissue kernel using a special software (SureSubtraction Ortho Version 6.0, Canon Medical Systems, or using the Vitrea enterprise 7.15 software) and were subtracted to obtain a bone free color‐coded CT‐S with a slice thickness of 3.0 mm in axial, coronal, and sagittal orientation. In this postprocessing technology is a registration process implemented to eliminate patient motion between both acquisitions. For patients whom subtraction could not be calculated because of severe motion artifacts, the iodine map from the postcontrast scan was instead generated using the CT console (dual‐energy raw data analysis, version 6.0, Canon Medical Systems, or Vitrea enterprise 7.15 software) and incorporated into the assessment of active joint inflammation.

### Image reading and survey

Two radiologists with different levels of experience (a senior consultant with 15 years [TD] and a trainee with 5 years [STU] of experience in musculoskeletal imaging) performed a radiologic assessment using all postprocessed images and individual series of the performed reconstructions. The trainee (STU) performed initial radiologic assessments, which were then validated by the senior consultant (TD), reflecting the common clinical practice. The aim of the assessment was to establish an imaging‐based suspected diagnosis based on individual imaging parameters, including structural and acute parameters of inflammation, using a semistructured report. Radiologists had access to the results of clinical examinations as well as laboratory tests. The imaging findings were subsequently presented and discussed in an interdisciplinary conference with the referring rheumatologists. The final diagnosis was determined by the referring rheumatologists, who considered the entire clinical picture, including both imaging findings and other diagnostic criteria, to establish the most accurate diagnosis for the patient.

Thereafter, the primary referring rheumatologists were asked to evaluate the diagnostic utility and impact of CE‐DECT on patient management using a questionnaire. The survey included the following questions: (1) what was the suspected diagnosis before study inclusion, (2) what was the final diagnosis after study inclusion, (3) how helpful was CE‐DECT for the final diagnosis (on a numeric rating scale of 1–10) and (4) how helpful was CE‐DECT for patient management (on a scale of 1–10)? Optional questions included (1) which aspects of CE‐DECT were especially helpful and (2) were there any complications or aspects that were not helpful?

### Statistical analysis

Statistical analysis was performed using GraphPad Prism (version 7 for MacOS, GraphPad Software). Imaging findings using CE‐DECT were juxtaposed with the initial and final diagnoses determined by the referring rheumatologists. This evaluation focused on surrogate performance criteria, emphasizing the added diagnostic value of CE‐DECT in detecting specific imaging biomarkers associated with various arthritic pathologies. Descriptive statistical analysis was conducted to summarize the frequency and distribution of key imaging findings, as well as to evaluate the agreement between CE‐DECT findings and the final diagnoses. Descriptive statistical analysis was performed to assess the results of the survey.

## RESULTS

### Patients

CE‐DECT was performed in 156 patients. In two patients, the CT‐S could not be calculated owing to severe motion between the pre‐ and postcontrast scans. Therefore, the iodine map was additionally reconstructed to assess active inflammation. In one examination, the scan was prematurely terminated at the patient's request before the application of the contrast agent and was therefore excluded from further analysis. In all other patients, CE‐DECT and the subsequent reconstructions algorithms were performed successfully. We observed a mild contrast agent adverse effect in one patient (nausea) after the scan. In 19 of these patients, the rheumatologists’ survey data were missing. Therefore, 136 patients (80 women) with a mean ± SD age of 58.1 ± 16.2 years (range, 18–87 years) were included in the analysis. The mean ± SD C‐reactive protein level was 22.6 ± 44.4 mg/L. Anticitrullinated protein antibody was positive (>20 IU/mL) in 34 patients, rheumatoid factor IgA (>20 IU/mL) in 19 patients, and rheumatoid factor IgM (>20 IU/mL) in 21 patients. The suspected clinical diagnosis without undergoing CE‐DECT included rheumatoid arthritis (RA) in 55 patients, psoriatic arthritis (PsA) in 18 patients, CPPD in 12 patients, OA in 14 patients, gout in 12 patients, and other diseases (eg, systemic sclerosis, systemic lupus erythematosus, myositis, and systemic vasculitis) in 29 patients.

After undergoing CE‐DECT, 69 patients were finally diagnosed with RA, 12 with PsA, 14 with CPPD, 7 with gout, 12 with OA, and 13 with other diseases. In nine patients, the findings were considered normal by the rheumatologists (Table 1). The average total dose‐length product was 93.2 mGy × cm resulting in an estimated effective dose of 0.075 mSv.

**Table 1:** Patient characteristics and CE‐DECT findings in patients’ levels*

	RA	PsA	CPPD	Gout	OA	Other	Normal
Patients, n (%)	69 (50.7)	12 (8.8)	14 (10.3)	7 (5.1)	12 (8.8)	13 (9.6)	9 (6.6)
Sex, women:men	36:33	9:3	10:4	2:5	8:4	9:4	6:3
Mean age (SD), y	59.7 (15.2)	49.8 (14.7)	73.4 (9.3)	64.3 (9.8)	62 (9.2)	43.2 (16.2)	44.2 (16.7)
CRP, mean (SD), mg/L	27.0 (48.2)	12.5 (26.1)	21.6 (58.8)	36.0 (54.1)	6.5 (9.5)	19.2 (30.4)	21.5 (53.5)
ACPA positivity, n/N	21/69	0/12	4/14	1/7	5/12	2/13	1/9
RF‐IgA positivity, n/N	15/69	1/12	0/14	1/7	0/12	2/13	0/9
RF‐IgM positivity, n/N	17/69	2/12	0/14	0/7	0/12	2/13	0/9
CE‐DECT imaging findings, n/N							
Synovitis	68/69	11/12	13/14	4/7	11/12	8/13	1/9
Tenosynovitis	47/69	3/12	6/14	4/7	0/12	4/13	0/9
BME	33/69	4/12	5/14	3/7	1/12	2/13	0/9
Erosion	30/69	3/12	5/14	4/7	0/12	3/13	0/9
Crystal	7/69	0/12	1/14	7/7	2/12	3/13	0/9
Periosteal proliferation	3/69	3/12	1/14	0/7	0/12	1/13	0/9
Ankylosis	7/69	2/12	0/14	0/7	0/12	0/13	0/9

* ACPA, anticitrullinated protein antibody; BME, bone marrow edema; CE‐DECT, contrast‐enhanced dual‐energy computed tomography; CPPD, calcium pyrophosphate dehydrate deposition disease; CRP, C‐reactive protein; OA, osteoarthritis; PsA, psoriatic arthritis; RA, rheumatoid arthritis; RF, rheumatoid factor.

### Image reading

CE‐DECT was positive for synovitis in 116 patients and for tenosynovitis/peritendonitis in 64 patients. Forty‐five patients had erosions, and 48 patients were positive for bone marrow edema. In eight patients, CE‐DECT showed periosteal proliferations indicating PsA. Crystals were detected in 20 patients, in 7 of these, tophus burden was identified using the uric acid reconstruction algorithm (Table 1).

**Figure 1:**
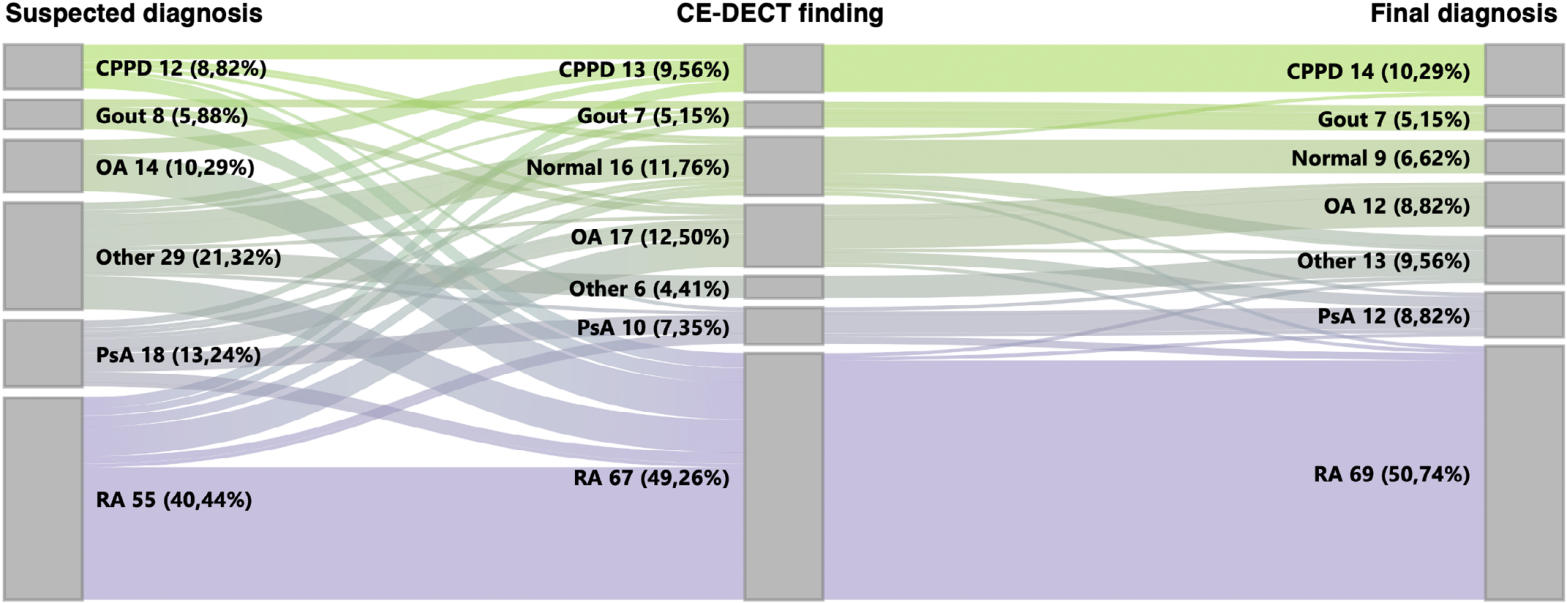
Comparison of the diagnoses. “Other” included patients with the final diagnosis of systemic sclerosis (n = 2), systemic lupus erythematosus (n = 1), unspecific arthritis (n = 2), idiopathic calcinosis (n = 1), Sjögren disease (n = 1), anti–tumor necrosis factor–induced paradox arthritis (n = 1), extraarticular manifestation of acne (n = 1), adult‐onset Still disease (n = 1), postoperative wound infection without joint involvement (n = 1), multicentric carpotarsal osteolysis syndrome (n = 1), and serum sickness (n = 1). Overall, in 119 patients (87.5%), the CE‐DECT finding aligned with the final diagnosis. In 67 patients (49.2%), CE‐DECT challenged the clinical suspected diagnoses and altered the final diagnosis. The greatest impact on the final diagnosis was observed with CE‐DECT in patients diagnosed with RA, where an additional 29 patients were ultimately diagnosed with RA based on imaging findings after the CE‐DECT, despite having a different clinical suspicion before the scan. Furthermore, no patient was incorrectly classified as positive for a disease in CE‐DECT who was ultimately deemed “normal” by rheumatologists. CE‐DECT, contrast‐enhanced dual‐energy computed tomography; CPPD, calcium pyrophosphate dehydrate deposition disease; OA, osteoarthritis; PsA, psoriatic arthritis; RA, rheumatoid arthritis.

**Figure 2:**
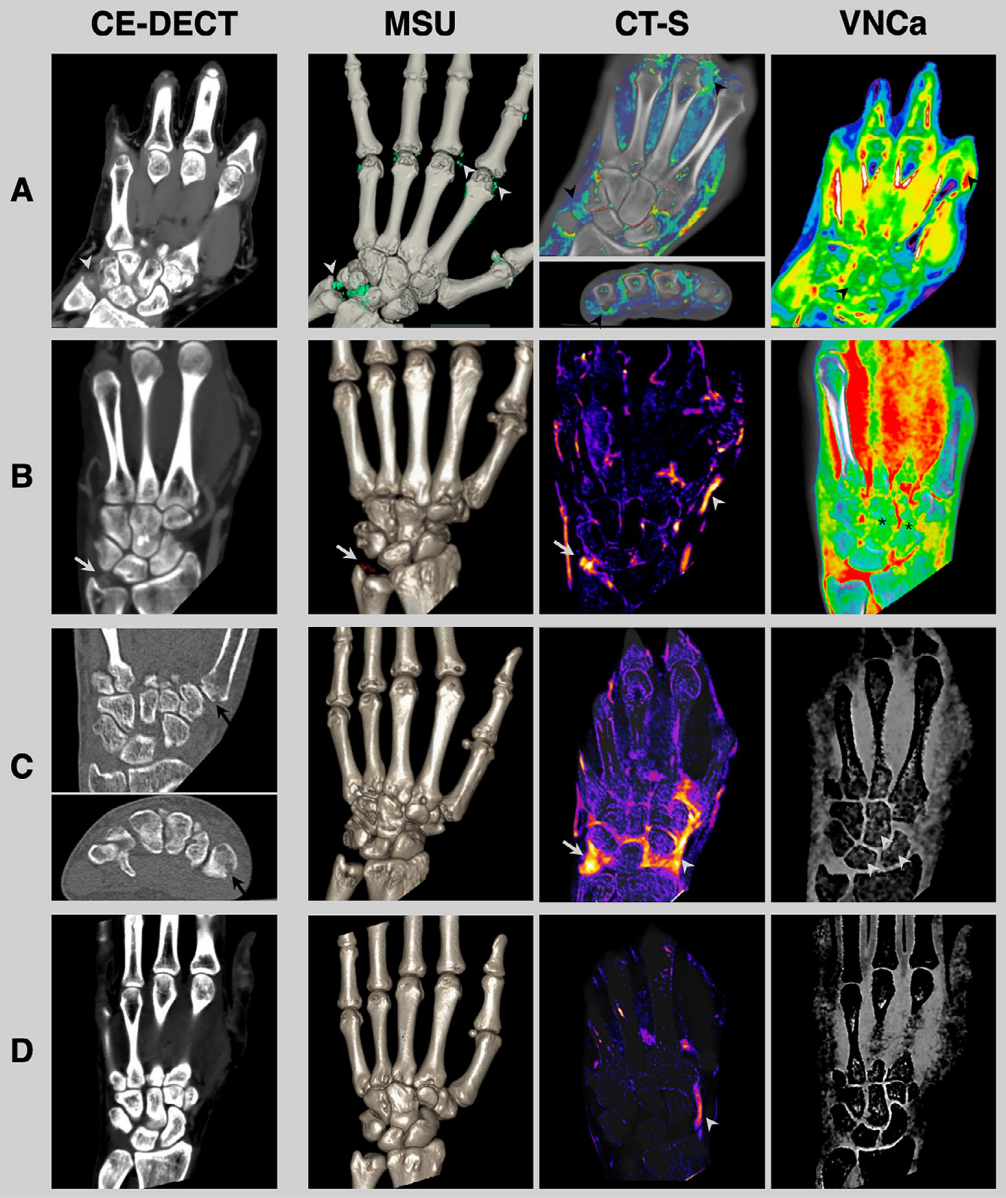
Imaging examples. (A) A 65‐year‐old male patient with an initially suspected diagnosis of psoriatic arthritis. In CE‐DECT, crystals were visible in the region of the triangular fibrocartilage complex (gray arrowhead) and the second and third metacarpophalangeal joint, which were confirmed to be MSU crystals. CT‐S showed synovitis of the carpus (including the region of the triangular fibrocartilage complex) at the metacarpophalangeal III and tenosynovitis of the flexor tendon V (black arrowheads). Color‐coded VNCa revealed BME of the carpal bones (black arrowhead), indicated by increased water content within the bone (increased brightness of the bone). Thus, CE‐DECT was able to refute the initial suspicion of psoriatic arthritis and help establish the final diagnosis of active tophaceous gouty arthritis. (B) A 66‐year‐old male patient with an initially suspected diagnosis RA. CE‐DECT revealed MSU crystals at the triangular fibrocartilage complex (white arrows, as indicated by red color coding), with active inflammation detected in CT‐S (white arrow). The white arrowhead in CT‐S indicates contrast medium within a vessel. Color‐coded VNCa showed BME in the scaphoid and capitate bones (*) indicated by increased brightness of the bone. Thus, CE‐DECT facilitated a change in the final diagnosis to gouty arthritis. (C) A 43‐year‐old female patient with an initially suspected diagnosis of gouty arthritis. CE‐DECT showed erosions at the base of the first metacarpal bone (black arrows). MSU reconstruction was negative for crystals. CT‐S demonstrated severe synovitis in the radiocarpal and intercarpal joints (white arrowhead and white arrow). Moderate BME was detected using non–color‐coded VNCa in the carpus (white arrowheads), indicated by increased brightness of the bone compared to, for example, the os triquetrum. Therefore, CE‐DECT findings were in line with the diagnosis of RA and did not support the diagnosis of gouty arthritis. Finally, the established diagnosis by the rheumatologist was RA. (D) A 30‐year‐old male patient with an initially suspected diagnosis of adult‐onset Still disease. No abnormalities were detected in CE‐DECT, leading to an assumption of a normal finding. However, the patient ultimately received a final diagnosis of adult‐onset Still disease. Contrast medium can be seen within vessels in CT‐S (white arrowhead) but not within the synovium. BME, bone marrow edema; CE‐DECT, contrast‐enhanced dual‐energy computed tomography; CT‐S, computed tomography subtraction to assess active inflammation in joints and tendons; MSU, monosodium urate reconstruction; RA, rheumatoid arthritis; VNCa, virtual noncalcium images to assess bone marrow edema. Color figure can be viewed in the online issue, which is available at http://onlinelibrary.wiley.com/doi/10.1002/art.43270/abstract.

The comparison among the diagnoses included (1) clinical suspected diagnosis before study inclusion, (2) imaging findings in CE‐DECT, and (3) the final diagnosis determined by the rheumatologists revealed that in 119 patients (87.5%), the CE‐DECT finding agreed with the final diagnosis, underscoring the impact of CE‐DECT on shaping the diagnosis. In 67 patients (49.2%), the initial clinical suspected diagnoses were altered following CE‐DECT (Figure 1). The greatest impact on the final diagnosis was observed with CE‐DECT in patients newly diagnosed with RA. Following CE‐DECT, 29 patients were newly diagnosed with RA, despite having different clinical suspicions before the scan. Similarly, the CE‐DECT results led to the diagnosis of more patients with CPPD (n = 9) and gout (n = 5), as initially suspected. Discrepancies between the CE‐DECT findings and the final diagnosis primarily occurred in patients who were classified as “normal” or diagnosed with OA based on imaging. Interestingly, among the 16 patients classified as “normal” in the CE‐DECT, 4 received a final diagnosis of “other.” Notably, three of these patients had already been clinically suspected of having an “other” diagnosis, suggesting that imaging did not provide significant additional insights in these cases. Furthermore, in 3 of 17 patients, for whom OA was suspected based on CE‐DECT findings, the final diagnosis changed to PsA. Importantly, no patient was misclassified as positive for a disease in CE‐DECT if they were ultimately deemed “normal” by rheumatologists. Imaging examples are shown in Figure 2.

**Figure 3:**
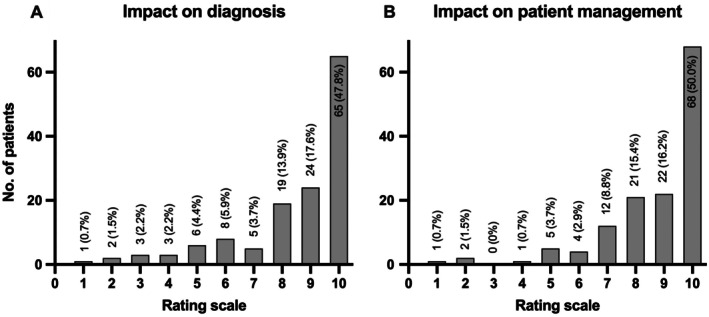
Results of the survey. (A) How helpful was contrast‐enhanced dual‐energy computed tomography for diagnosis? 1 = not helpful to 10 = very helpful. (B) How helpful was contrast‐enhanced dual‐energy computed tomography for patient management? 1 = not helpful to 10 = very helpful.

Rheumatologists rated CE‐DECT's diagnostic utility at mean ± SD of 8.5 ± 2.1 and its contribution to patient management at 8.7 ± 1.8 (Figure 3). The ability of CE‐DECT in differential diagnostic evaluation, as well as the clear findings and reliable assessment of acute inflammatory and structural lesions, were considered particularly helpful by the rheumatologists. Furthermore, no complications arising from the CE‐DECT examination were reported by the rheumatologists.

## DISCUSSION

To the best of our knowledge, this is the first study investigating the clinical impact of CE‐DECT in patients with suspected inflammatory arthritis of the hand. CE‐DECT showed a significant influence on diagnosis, which can decisively affect further patient management. In 119 patients (87.5%), the CE‐DECT findings agreed with the final diagnosis. In approximately 50% of the included patients, the initially suspected diagnosis was changed following CE‐DECT. This highlights the potential impact of CE‐DECT in the diagnostic workup in arthritis, as it can provide crucial additional information and, thus, cover a broad differential diagnostic spectrum. The CE‐DECT findings provide the greatest benefit for patients with RA and are particularly valuable for the definitive detection of different crystal arthropathies. This capability is also reflected in its utility for further patient management.

The use of imaging in detecting and differentiating arthritis holds significant importance, with the choice of appropriate imaging modalities depending on various factors, including existing infrastructure and patient‐specific considerations.^22^ Ultrasound is typically employed as the initial imaging modality and is often performed by experienced rheumatologists. This technique allows for the accurate visualization of active inflammation in joints and tendons^23^; however, its diagnostic performance is significantly dependent on the examiner's expertise, and the results may lack objective quantification.^24^, ^25^ Also, ultrasound is not routinely recommended for therapeutic monitoring, as current evidence suggests that it does not provide substantial additional value beyond clinical examination.^26^ In contrast, conventional x‐rays primarily serve to distinguish between differential diagnoses during initial assessments and establish a baseline before treatment initiation, helping to exclude any existing structural lesions. A notable limitation of x‐rays lies in their low sensitivity and specificity, as they are unable to evaluate active inflammation effectively.^27^ MRI is no longer recommended for initial imaging of early arthritis in current guidelines owing to its limited specificity and is therefore primarily reserved for more complex cases.^26^ This situation engenders a diagnostic gap in the evaluation of arthritis, highlighting the need for more effective imaging strategies.

CE‐DECT provides advantages and additional information,^28^ establishing it as a particularly valuable and influential imaging modality in the diagnosis of arthritis. Its multiparametric capabilities facilitate the comprehensive assessment of relevant information related to structural changes, active inflammation, and differential diagnoses with a high degree of reliability.^6^, ^29^, ^30^, ^31^, ^32^ Thus, a single examination can yield extensive information that substantially enhances the detection and differentiation of various forms of arthritis. This comprehensive assessment may reduce the need for multiple separate imaging examinations, thereby streamlining the diagnostic process in selected cases in which differential diagnosis is challenging. The results of this study contribute critical evidence supporting the incorporation of CE‐DECT into routine clinical practice, underscoring its significant role in facilitating accurate diagnosis. The greatest impact on the final diagnosis was primarily observed in patients with RA and for the detection of crystal arthropathies. The capability for DECT‐specific material differentiation is crucial for the diagnosis of crystal arthropathies, which is not achievable with any other imaging technique. Another contributing factor is its markedly enhanced ability to reliably assess subtle structural lesions, such as erosions. However, in cases of rare diseases with joint manifestations, CE‐DECT encounters limitations, possibly because of the absence of “typical” imaging parameters compared with other types of arthritis. It is noteworthy that it has been consistently demonstrated that CE‐DECT is effective in differentiating between healthy individuals and those with disease. A notable advantage of CE‐DECT is its improved availability compared with MRI, particularly in nonindustrialized regions. Concerns about radiation exposure are mitigated by an estimated effective dose of CE‐DECT comparable to that of conventional x‐ray of the hands.^12^ Furthermore, patients often exhibit a preference of CT over MRI owing to its rapid availability and significantly shorter scan durations.^11^ This preference further emphasizes the potential for CE‐DECT to improve patient management within clinical settings.

This study was explicitly designed to investigate the impact of CE‐DECT within a clinical setting for patients with suspected arthritis. One limitation of the study is that no direct comparison was made with MRI and/or ultrasound or clinical disease activity parameters, as not all patients underwent MRI or ultrasound as part of their standard clinical assessment and these comparisons have already been undertaken.^11^, ^12^ The radiologists involved in the study were not blinded to clinical and laboratory findings, which aligns with typical clinical practice.^33^ The study was conducted at a single center, which may limit the generalizability of the results to other health care institutions. Consequently, further investigation into the clinical significance of CE‐DECT across multiple centers is essential to enhance the generalizability of these findings. Although DECT is becoming more widespread, a further limitation concerns the availability of DECT systems and the reconstruction of advanced images, which is currently vendor‐dependent and not yet universally accessible. Therefore, broader implementation of CE‐DECT in clinical routine will depend on future technical standardization and multicenter validation.

A further potential limitation is the involvement of different rheumatologists involved in the process of making the diagnosis. Differences in clinical assessment and diagnostic criteria between observers may introduce variability. In this context, interobserver variability was not systematically analyzed, which could influence the consistency of diagnoses. The clinical relevance of an imaging modality in the diagnosis of arthritis extends beyond initial assessment to include its application in follow‐up imaging for evaluating treatment efficacy and monitoring changes during therapy; however, these aspects were not addressed in this study. We also did not include an additional assessment months or years after initial diagnosis to see whether findings are still in line with the established diagnosis. Although CE‐DECT provides valuable multiparametric information, future studies should also investigate the diagnostic utility of noncontrast DECT protocols, particularly for patients with contraindications to contrast media or limited access to contrast administration.

In our investigation, we have shown that CE‐DECT had a significant impact on the diagnosis and management in patients with suspected arthritis. CE‐DECT offers standardized arthritis imaging and, because of various reconstruction methods, allows the acquisition of more varied yet standardized information.^6^, ^11^, ^12^ Compared with sonography and MRI, it may therefore represent a promising complementary one‐stop‐shop technique in arthritis imaging. In summary, the integration of advanced imaging techniques like CE‐DECT into routine clinical practice not only enhances diagnostic accuracy but also supports improved therapeutic decision‐making in the management of rheumatic diseases, with future multicenter studies needed to further validate these findings.

## AUTHOR CONTRIBUTIONS

All authors contributed to at least one of the following manuscript preparation roles: conceptualization AND/OR methodology, software, investigation, formal analysis, data curation, visualization, and validation AND drafting or reviewing/editing the final draft. As corresponding author, Dr Ulas confirms that all authors have provided the final approval of the version to be published and takes responsibility for the affirmations regarding article submission (eg, not under consideration by another journal), the integrity of the data presented, and the statements regarding compliance with institutional review board/Declaration of Helsinki requirements.

## Supporting information


**Disclosure form**.
